# Three-Stage Pavement Crack Localization and Segmentation Algorithm Based on Digital Image Processing and Deep Learning Techniques

**DOI:** 10.3390/s22218459

**Published:** 2022-11-03

**Authors:** Zhen Yang, Changshuang Ni, Lin Li, Wenting Luo, Yong Qin

**Affiliations:** 1 College of Transportation and Civil Engineering, Fujian Agriculture and Forestry University, Fuzhou 350108, China; 2 College of Transportation Engineering, Nanjing Tech University, Nanjing 211816, China; 3 School of Traffic and Transportation, Beijing Jiaotong University, Beijing 100044, China

**Keywords:** digital image processing technology, asphalt pavement crack, deep learning, guided filter, Retinex, YOLOv7, attention mechanism

## Abstract

The image of expressway asphalt pavement crack disease obtained by a three-dimensional line scan laser is easily affected by external factors such as uneven illumination distribution, environmental noise, occlusion shadow, and foreign bodies on the pavement. To locate and extract cracks accurately and efficiently, this article proposes a three-stage asphalt pavement crack location and segmentation method based on traditional digital image processing technology and deep learning methods. In the first stage of this method, the guided filtering and Retinex methods are used to preprocess the asphalt pavement crack image. The processed image removes redundant noise information and improves the brightness. At the information entropy level, it is 63% higher than the unpreprocessed image. In the second stage, the newly proposed YOLO-SAMT target detection model is used to locate the crack diseases in asphalt pavement. The model is 5.42 percentage points higher than the original YOLOv7 model on mAP@0.5, which enhances the recognition and location ability of crack diseases and reduces the calculation amount for the extraction of crack contour in the next stage. In the third stage, the improved k-means clustering algorithm is used to extract cracks. Compared with the traditional k-means clustering algorithm, this method improves the accuracy by 7.34 percentage points, the true rate by 6.57 percentage points, and the false positive rate by 18.32 percentage points to better extract the crack contour. To sum up, the method proposed in this article improves the quality of the pavement disease image, enhances the ability to identify and locate cracks, reduces the amount of calculation, improves the accuracy of crack contour extraction, and provides a new solution for highway crack inspection.

## 1. Introduction

At present, according to different detection objects, pavement disease detection technology can be divided into two kinds. The first is laser displacement detection technology, which mainly takes pavement deformation diseases as the detection object. Through three-dimensional processing, relevant index data are obtained, and then the damage degree of pavement rutting, subsidence, and other diseases are evaluated. The other is the more commonly used digital image detection technology. It mainly takes pavement crack disease as the detection object, collects high-definition image data of the pavement through shooting, and then uses image processing and other methods to obtain relevant information such as pavement cracks.

Different from the detection of pavement deformation, the demand for pavement crack detection is large, which is more common in pavement quality detection and maintenance management. When the pavement structure enters the early stage of degradation, pavement cracks are formed. If pavement cracks continue to develop, the damage degree of the pavement will be further aggravated. Before the 1970s, the traditional pavement crack detection methods involved manual detection: not only the need to record the length, width, and severity of the crack, but also the need to draw the crack location map. In addition, limited by the experience of pavement crack detection personnel, the test results are also susceptible to subjective impact. With the development of digital image technology and computer technology, there is more and more research on the automatic detection of pavement cracks, especially in the fields of image enhancement, image segmentation, and image recognition of pavement cracks.

In the image preprocessing algorithm, the image enhancement algorithm plays a very important role. The image enhancement algorithm highlights the useful information in the image while removing the unimportant information in the image. The image enhancement algorithm can not only improve the visual effect of the image but also make the subsequent image processing work more convenient. When the vehicle-mounted camera is used to obtain the road image, factors such as uneven illumination distribution, environmental noise, occlusion shadows, and foreign bodies on the road will affect the quality of the road image and interfere with the information extraction of subsequent road disease images. Therefore, in the road image preprocessing stage, image enhancement processing must be carried out to eliminate the influence of interference factors.

To eliminate the influence of different interference factors on pavement crack image quality, many scholars have done relevant research. For example, for uneven illumination distribution, Cheng H D et al. subtracted the original road surface image from the blurred image after low-pass filtering to obtain an image difference [1]. This not only eliminates the impact of light changes on the pavement crack image but also retains the crack information, to some extent, reducing the tire traces, white lines on the pavement, and other noise. For the influence of environmental noise in pavement crack images, Zuo Y et al. also proposed new methods from different angles. For example, Zuo Y et al. added the wavelet decomposition algorithm to the pavement crack image enhancement method [2]. The algorithm decomposes the pavement image, then reduces the noise of each scale, and finally achieves the purpose of reducing the noise in the pavement crack image. In addition, many researchers have also applied fuzzy logic (FL) to pavement crack image enhancement methods. For example, Bhutani K R et al. proposed a pavement crack image enhancement method based on FL through experiments [3].

To extract the characteristics of pavement crack images more conveniently, image segmentation is also needed based on image enhancement. Image segmentation divides the image into several specified regions according to the characteristics of different regions in the image. For pavement crack images, the image can be divided into the background region and the crack region. At present, segmentation algorithms based on threshold, edge detection, region, and FL are commonly used image segmentation algorithms.

Threshold segmentation is an algorithm for image segmentation by setting a threshold, which is mostly used in the field of image segmentation. However, there are still many problems in determining the threshold for different images. Therefore, relevant scholars have also done a lot of research on pavement crack images. For example, Kirschke K et al. divided the image into multiple sub-blocks and used the gray histogram to perform threshold segmentation of pavement crack images [4], but the accuracy was general. Combining morphology and maximum entropy, Oliveira H et al. performed dynamic threshold segmentation on pavement crack images [5]; on the other hand, Cheng et al. proposed two threshold segmentation algorithms for pavement crack images based on FL and sample space. One method is to obtain the global threshold by FL, then binarize the image difference obtained by subtracting the mask image from the original image, and finally realize the segmentation [6]. Another method is to determine the threshold by reducing the sample space and interpolation, using the mean and variance of pixel gray to achieve real-time threshold segmentation [7]. The segmentation results of these two methods are still insufficient, the false detection rate is high, and there are isolated noise points on the edge of the crack. At the same time, the active contour model (ACM) has good segmentation accuracy, so it is often used in the field of image segmentation. However, when dealing with images with uneven intensity and more noise, the method will be extremely unstable. In addition, the calculation process of most existing ACMs is complex, which makes it time-consuming and inefficient. Ge et al. proposed an active contour approach driven by adaptive local pre-fitting energy function based on Jeffreys divergence (APFJD) for image segmentation. Although the calculation process of the model is also very complicated, Ge designed a pre-fitting function calculated before the iteration process, which reduces a lot of calculation time and improves the segmentation accuracy. Intensity inhomogeneity brings great difficulties to image segmentation [8]. Weng proposed an additive bias correction (ABC) model based on intensity inhomogeneity. Compared with the traditional image segmentation model, this model has stronger robustness, faster speed, and higher accuracy [9]. Ge et al. proposed a hybrid active contour model driven by pre-fitting energy with an adaptive edge indicator function and an adaptive sign function. The key idea of employing the pre-fitting energy is to define two pre-fitting functions to calculate mean intensities of two sub-regions separated from the selected local region based on pre-calculated median intensity of the selected local region before the curve evolves, which saves a huge amount of computation cost [10].

In the crack image segmentation method based on edge detection, the morphological method is more common. For example, through morphological, Sobel, and other methods, Tanaka et al. successfully segmented the pavement crack image, but the adaptability is poor and cannot segment small cracks [11].

At present, due to the lack of unified and open-source pavement crack image samples and determined algorithm evaluation criteria, the research on pavement crack image recognition algorithms by relevant scholars varies from region to region, and the universality is not strong. In addition, there are few studies on how to evaluate the degree of pavement crack damage. The use of image processing methods to identify pavement crack images is more traditional and classic. For example, Huang Z et al. preprocessed and detected pavement crack images with gray image edge detection, threshold classification, Sobel filtering, and the Otsu method [12]. Mathavan S et al. used the Gabor filter for pavement crack image recognition. Through the convolution of the filter and the preprocessed image, the binary output image is generated by thresholding, and then the generated binary image is combined to output the identified crack image [13]. The recognition effect of these two methods is general and the efficiency is not high. SONG Hong-xun et al. studied crack image recognition algorithms from the perspective of ridge edge detection. In terms of noise elimination, the multi-scale reduction of image data and threshold processing are combined to smooth the image while enhancing the cracks [14].

Wavelet transform can also be used as a means of crack location. The wavelet transform of mode shapes is widely used for the localization of cracks in beams and structures [15,16,17,18]. Wavelet transforms have excellent properties to extract the localized information in the of measurement noise [19]. Kumar and Singh [20] used the continuous wavelet transform (CWT) to locate the crack in a beam. Nigam and Singh [21] used discrete wavelet transform to detect the crack in a beam. Kumar et al. [22] studied the selection of suitable mother wavelets and the corresponding vanishing moments for the efficient localization of cracks.

At the same time, some famous scholars have used advanced mathematical methods to locate and detect cracks. Ramnivas Kumar et al. proposed a variance-based crack detection and localization method in beams [23]. Sara Nasiri et al. used data-driven technology to predict the fatigue of metals, composites, and 3D-printed parts [24].

With the increasing mileage of expressways at home and abroad, the existing means of using digital image processing technology to identify cracks have been unable to meet the needs of daily road inspection. At the same time, intelligent detection algorithms based on artificial intelligence and machine learning and deep learning have developed rapidly and have made good progress in the field of pavement crack identification [25].

Recently, damage in a cracked beam was detected by FL technique [26]. In fact, the FL approach is used to find the location and depth of cracks on a cantilever beam. On the fuzzy mechanics in crack detection, researchers used the FL control method, which is used in their previous research [27,28].

At present, many experts and scholars have carried out research on pavement crack image recognition through neural networks. For example, in the field of traditional machine learning, Oliveria et al. designed a classifier to classify pavement crack images using training methods [29].

In addition, in the field of deep learning, Lee B J et al. designed three neural network algorithms based on image grayscale, histogram, and neighbor points to automatically classify and recognize pavement crack images. The results show that the neural network algorithm based on neighbor points has a better recognition effect [30]. A convolutional neural network can improve performance by improving its structure. Zhang et al. designed CrackNet based on a convolutional neural network to identify asphalt pavement cracks. Compared with other network structures, the pooling layer of each output in this network structure is not reduced to ensure the quality of the image [31]. Compared with the crack recognition method based on machine learning, CrackNet has obvious advantages in accuracy. Han et al. [32] developed a semantic segmentation network that can reach the pixel level. Li et al. [33] proposed a feature fusion network based on Faster R-CNN to detect cracks on the arc top of alpine tunnels. Ju Huyan et al. [34] proposed a new feature fusion network for crack detection in complex backgrounds. In addition, Malini et al. [35] used a series of regularization methods to improve the performance of convolutional neural network models. Cha et al. [36] calculated the defect characteristics of concrete cracks based on machine vision and deep learning network architecture. Mogalapalli et al. [37] solved various image classification tasks based on quantum transfer learning. Pang et al. [38] proposed a new crack extraction method to solve the problem of noise and brightness in the image. Sekar et al. [39] identified and located fractures based on region of interest and global average pooling operation. The above methods based on convolutional neural network and deep learning have surpassed the traditional digital image processing methods in the speed of recognizing crack diseases, but their accuracy has not met the industrial demand.

The main contributions of this article are as follows:

(1) This article proposes a new image preprocessing method based on guided filtering and Retinex. Compared with traditional digital image detection technology, this method can eliminate the influence of uneven illumination distribution, environmental noise, occlusion shadow, and other external factors on image quality.
(2) To reduce the amount of calculation and extract the crack features in a targeted manner, this article proposes an improved target detection algorithm based on the parameter-free attention module SimAM and Transformer. The purpose of the algorithm is to accurately locate the area where the cracks exist in the image, thus, reducing redundant calculations for the next crack contour feature extraction. Compared with the existing convolutional neural network and deep learning methods, this algorithm enhances the accuracy of frame selection for the crack target area.
(3) In this article, the traditional k-means clustering algorithm is improved. The image noise is eliminated with Gaussian filtering, and the image pixel value is optimized so that the crack contour can be extracted more accurately.

**Figure 1:**
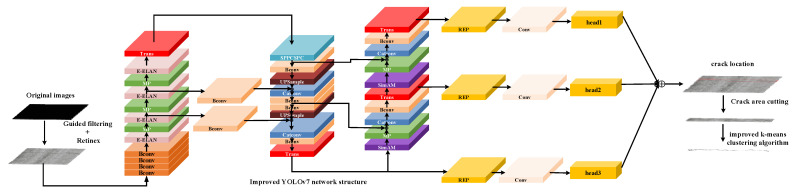
The network structure diagram of the proposed method.

The overall processing flow of this article is shown in Figure 1.

## 2. Materials and Methods

**Figure 2:**
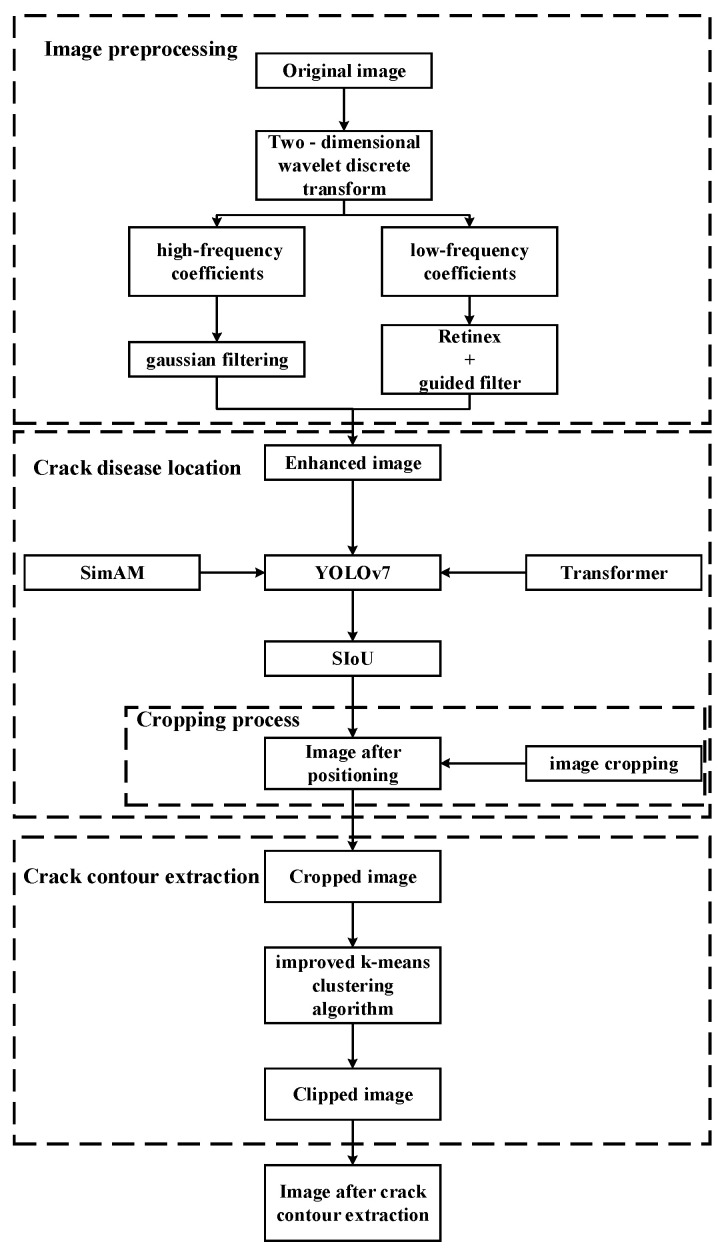
The flowchart of the proposed method.

In this study, the overall framework of the proposed method is mainly composed of the following steps: (1) image preprocessing, (2) crack disease location, and (3) crack contour extraction. A flow chart summarizing the process is presented in Figure 2.

As shown in Figure 2, this article is mainly divided into three stages to process the image data containing crack diseases. In the first stage, the image data is preprocessed by guided filtering and Retinex. This method first uses a two-dimensional discrete wavelet transform to denoise and compress the image, then uses the method of combining guided filtering with MSRCR to process the low frequency coefficients of wavelet, and finally uses soft threshold filtering to process the high frequency coefficients of the wavelet. In the second stage, the improved target detection model YOLO-SAMT is used to locate the crack disease. The target detection algorithm combines the non-parametric attention mechanism SimAM and Transformer, which can quickly locate the cracks in batches. At the same time, the located crack image data is cropped to remove redundant data for the next stage of crack extraction; in the third stage, a new crack contour extraction algorithm based on k-means clustering algorithm proposed in this article is used to accurately extract the crack contour.

### 2.1. Asphalt Pavement Image Enhancement Method

At present, the enhancement methods for crack images mainly include the following: the first method is the histogram equalization method, which can directly enhance the contrast of the image and bring about changes in the senses; the second method is the Retinex algorithm. The current Retinex algorithm includes SSR [40], MSRCR [41], SRIE [42], LIME [43] and other methods. The third method uses Gaussian filtering [44,45,46], bilateral filtering [47], guided filtering [48] and other methods to filter and denoise crack images. Histogram equalization has a good image enhancement effect. It can also be seen from the implementation algorithm that the main advantage is that it can automatically enhance the contrast of the entire image, but the specific enhancement effect is not easy to control, and only the histogram of the global equalization processing can be obtained. However, in actual operation scenarios, it is often necessary to process the local features of the image, so this method is not universal. The Retinex algorithm can better preserve the details of the image, and the processed image has moderate brightness and high contrast, but the image is prone to halo in the case of uneven lighting, resulting in blurred images. The biggest advantage of guided filtering is that it can use a linear function to calculate the output value of pixels, while bilateral filtering needs to consider factors such as the geometric characteristics and intensity of pixels. When processing larger images, the amount of computation will increase significantly.

Considering the advantages and disadvantages of the above-preprocessing methods, this paper proposes a new image enhancement method based on the Retinex algorithm and guided filtering based on wavelet transform. This method enhances the contrast of the image, overcomes the problem of loss of details after image enhancement, makes the optimized image clearer, and provides a rich database for subsequent disease identification and localization.

#### 2.1.1. Two-Dimensional Discrete Wavelet Transform

Two-dimensional discrete wavelet transform can denoise and compress images. Given scale function λ and wavelet function σ, one two-dimensional scale function and three two-dimensional wavelet functions can be combined [49]. The two-dimensional scale function is shown in Equation (1) As shown, the three two-dimensional wavelet functions are shown in Equations (2)–(4)

(1) λ(x,y)=λ(x)λ(y)

(2) $$\sigma^{H}\left(x,y\right)=\sigma \left(x\right)\lambda \left(y\right)$$

(3) $$\sigma^{V}\left(x,y\right)=\sigma \left(x\right)\lambda \left(y\right)$$

(4) $$\sigma^{D}\left(x,y\right)=\sigma \left(x\right)\sigma \left(y\right)$$

These wavelets measure the changes in grayscale in different directions in the image: $\sigma^{H}\left(x,y\right)$ represents the change of gray value along a column (such as a horizontal edge), $\sigma^{V}\left(x,y\right)$ represents the change of gray value along a row (such as a vertical edge), and $\sigma^{D}\left(x,y\right)$ represents the change of gray value along the diagonal.

**Figure 3:**
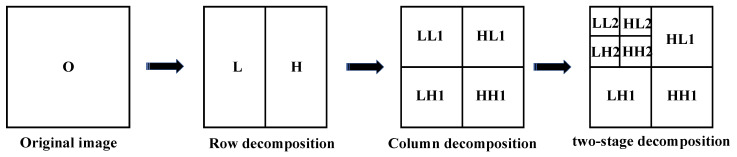
Flow chart of two-dimensional wavelet transform.

The flow chart of the two-dimensional wavelet transform of the image is shown in Figure 3:

The horizontal parameters of the original image O, which are low-frequency component L and high-frequency component H, can be obtained by performing one-dimensional wavelet transform on each row of the image; then, a one-dimensional wavelet transform is performed on each column of the transformed image O (L,H), and the parameters in the horizontal and vertical directions of the original image can be obtained; that is, the low-frequency component LL and the high-frequency component HH. At the same time, the high-frequency component LH and the low-frequency component HL can be obtained in the cross direction. HH represents the horizontal and vertical high-frequency components, indicating the details of the diagonal direction. LH represents horizontal low-frequency and vertical high-frequency parameters that indicate detailed information in the vertical direction. HL represents the horizontal high-frequency and vertical low-frequency parameters, which indicate the horizontal detail information.

#### 2.1.2. Processing Method of Wavelet Low-Frequency Coefficients

In Section 2.1.1, the original image is subjected to a two-dimensional wavelet transform operation to obtain the corresponding low-frequency coefficients and high-frequency coefficients. To be able to perform multi-scale analysis of the image, the low-frequency coefficients of the wavelet need to be processed. Different from the general image, the edge feature details of the pavement crack image are retained and are key. To better extract the information of the crack, we use the guided filter to process the image [50]. The guided filter can be used as an edge smoothing operator like a bilateral filter, but it has a better effect near the edge. In addition, regardless of the kernel size and intensity range, the guided filter has a fast and non-approximate linear time algorithm.

However, while processing the low-frequency coefficients of the wavelet, the problem of color distortion of the image becomes more and more prominent. To solve this problem, we select the MSRCR algorithm to restore the image color. MSRCR is based on MSR, by adding a color recovery factor to solve the problem of image distortion. We combine it with guided filtering to create a color restoration algorithm based on guided filtering theory. The implementation formula of the algorithm is shown in Equation (5):

(5) $$R_{MSRCR_{i}}{\left(x,y\right)}^{\prime }=a_{k}\left(G\cdot R_{MSRCR_{i}}\left(x,y\right)+O\right)+b_{k}$$

where $R_{MSRCR_{i}}{\left(x,y\right)}^{\prime }$ is the output image, $R_{MSRCR_{i}}\left(x,y\right)$ is the input image, G is the gain, O is the bias, and a and b are the two constants of the function when the center of the image is k.

#### 2.1.3. Wavelet High-Frequency Coefficient Soft Threshold Filtering Processing

The wavelet high-frequency coefficients decomposed in the previous stage contain some edge information, noise information, and other details of our image, and the use of threshold filtering can often effectively remove excess noise.

Our common threshold filtering methods are divided into soft threshold filtering, hard threshold filtering, and half-threshold filtering. Among them, the advantage of hard threshold filtering is that the signal-to-noise ratio is high, but the processed images are often seriously distorted; soft threshold filtering can smooth the image details and effectively improve the distortion; half-threshold filtering has the best smoothing effect, but the amount of calculation is large. To sum up, we employ soft threshold filtering to deal with wavelet high-frequency coefficients.

The expression of soft threshold filtering is shown in Equation (6).

(6) $$G_{T}=\left\{ \begin{array}{l} \left[\mathrm{sgn}\left(g\right)\right]\left(\left|g\right|-T\right),\left|g\right|\geq T\\ 0,\left|g\right|<T \end{array}\right.$$

where $G_{T}$ is the processed high-frequency coefficient, g is the high-frequency coefficient, T is the threshold, $T=\sqrt{2\log_{2}\left(l\right)\partial }$, l is the signal length, and ∂ is the noise variance

#### 2.1.4. Experimental Results and Analysis

**Figure 4:**
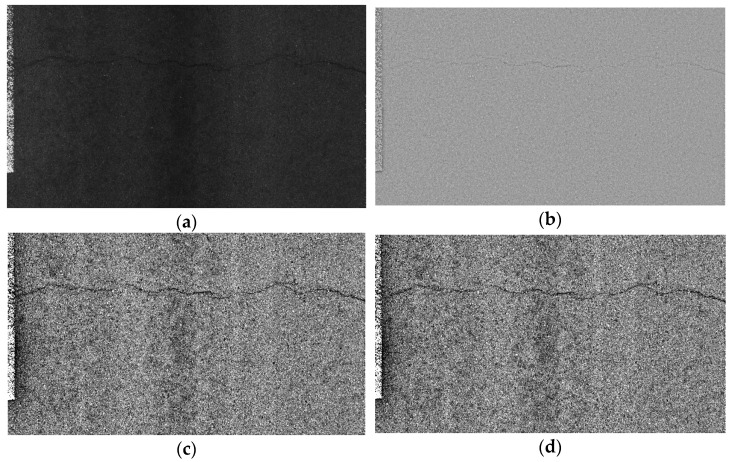

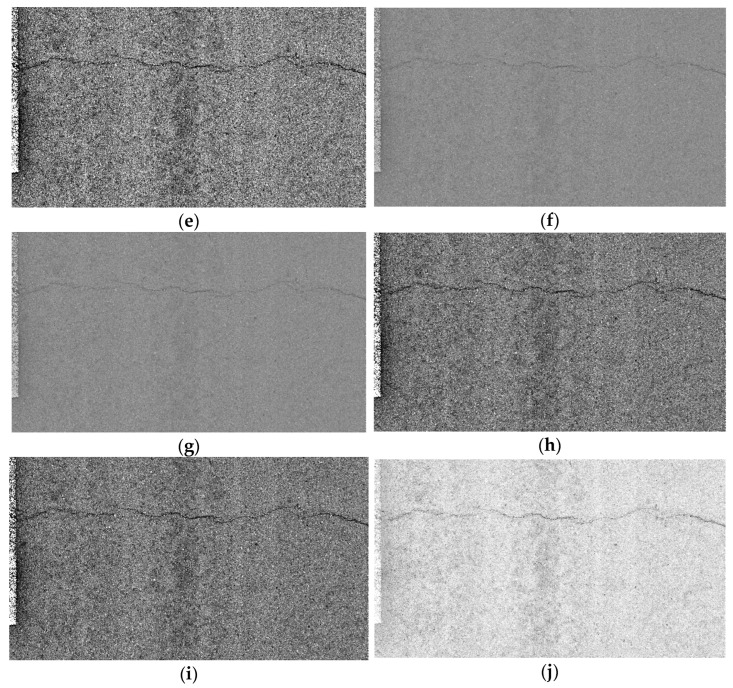
The results of various algorithms for crack disease image processing. (**a**) Original image, (**b**) SSR, (**c**) AutoMSRCR, (**d**) OpenCV, (**e**) MATLAB, (**f**) Gimp, (**g**) MSR, (**h**) MSRCP, (**i**) MSRCR, (**j**) method proposed in this paper.

To verify the practicability of the image enhancement method proposed in this section, a comparative experiment on image processing of asphalt pavement cracks was carried out; algorithms such as SSR, AutoMSRCR, OpenCV, Matlab, Gimp, MSR, MSRCP, and MSRCR were selected; and the images proposed in this section were respectively selected. The processing methods are compared, and the schematic diagram of various algorithms processing crack disease images is shown in Figure 4.

As a qualitative evaluation index, information entropy is often used in the evaluation of image quality. The higher the information entropy [51], the better the image quality, and more information can be obtained from the image. Its expression is shown in Equation (7).

(7) $$H\left(p\right)=-\sum_{i=0}^{255}p_{i}\log_{2}p_{i}$$

where $p_{i}$ represents the probability of occurrence of the i-th gray level. The information entropy of various algorithms for processing crack disease images is shown in Table 1.

The traditional image enhancement methods focus on adjusting the color or brightness of the image but ignore image denoising. The method proposed in this article firstly performs the two-dimensional discrete wavelet transform on the image to obtain the low-frequency and high-frequency coefficients of the wavelet. For the processing of wavelet low-frequency coefficients, this article uses the method of combining guided filtering with the MSRCR algorithm to extract multi-scale information of the image while preserving the color of the image. For the processing of wavelet high-frequency coefficients, this article selects soft threshold filtering for denoising. From Table 1, the information entropy of the crack disease image processed by this method is higher than that of other common algorithms, which quantitatively proves the superiority of the image processing algorithm in the proposed method.

### 2.2. Crack Disease Location Based on Improved YOLOv7

#### 2.2.1. YOLOv7 Network Architecture

**Figure 5:**
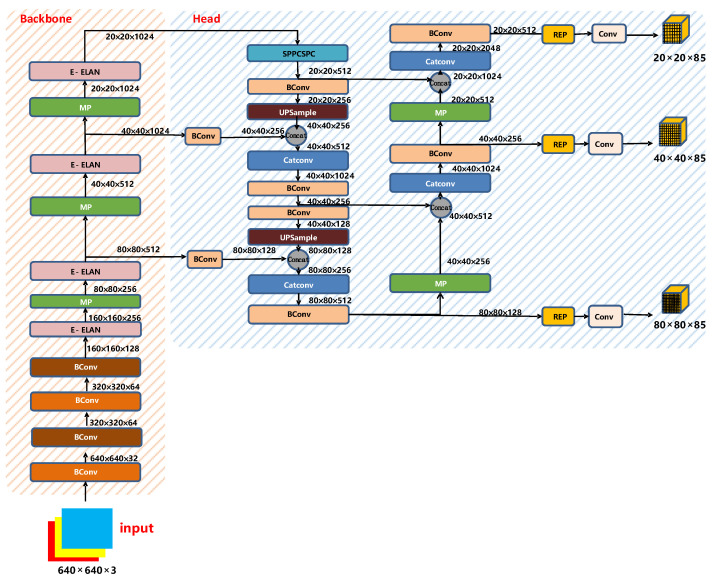
YOLOv7 network structure diagram.

The YOLO series of target detection algorithms are faster and more accurate than synchronous algorithms [52,53,54,55]. In 2022, YOLOv7 was formally applied to target detection [56]. Based on image processing of asphalt pavement crack disease, this paper uses YOLOv7 to further locate cracks. The network structure diagram of YOLOv7 is shown in Figure 5.

1. Input terminal

The preprocessing method of YOLOv7 is similar to YOLOv5, such as the mosaic method, adaptive anchor box, and adaptive image scaling.

In the network training stage, YOLOv7 uses the Mosaic data enhancement method, which is improved with the CutMix data enhancement method. CutMix uses only two images, while Mosaic’s data enhancement method uses four images that are randomly scaled, cropped, and arranged. This enhancement method can combine several images into one image, which greatly improves the training speed of the network and reduces the memory requirement of the model, while increasing the diversity of the dataset, and also increases the detection accuracy of the network.

2. Backbone

**Figure 6:**
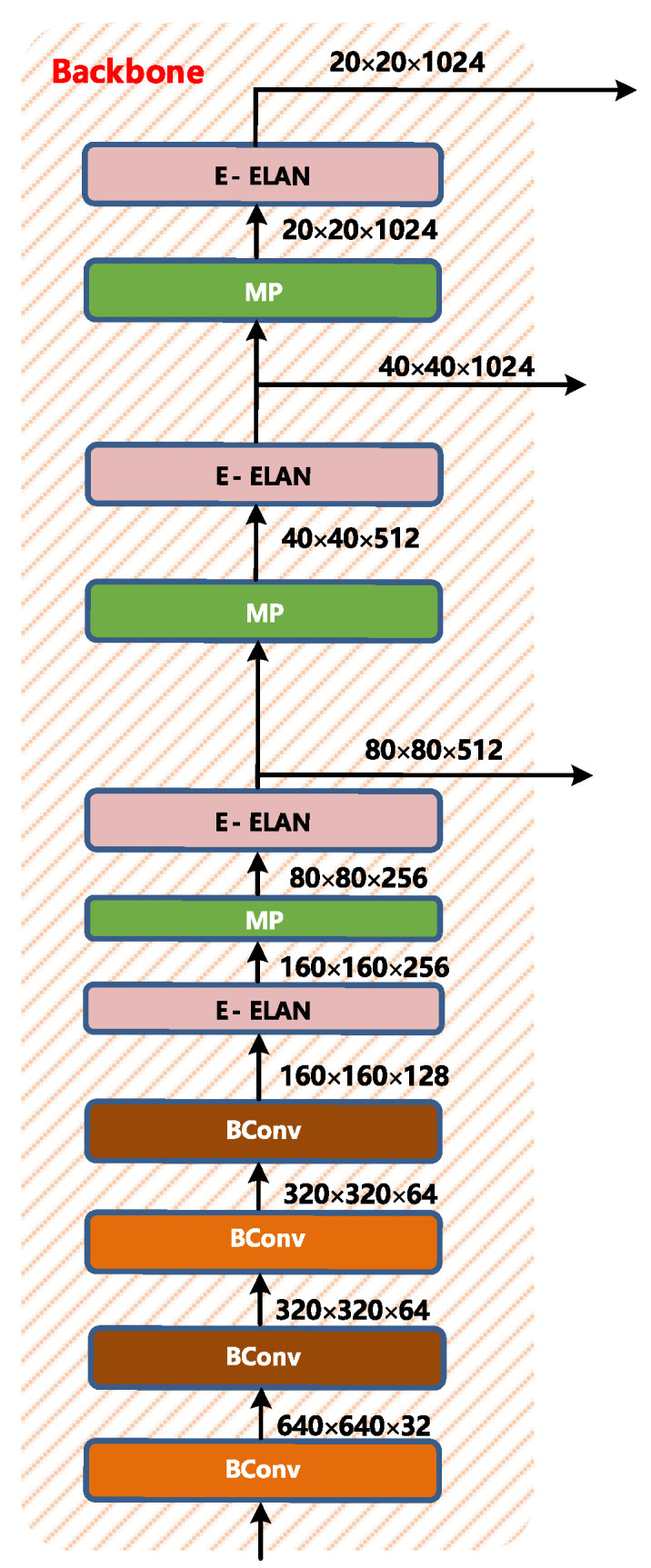
Backbone structure of YOLOv7.

**Figure 7:**
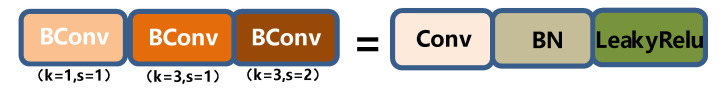
BConv structure layer diagram.

The backbone layer of YOLOv7 is shown in Figure 6. It is composed of several Bconv layers, E-ELAN layers, and MP layers. The BConv layer consists of the convolution layer, BN layer, and activation function. The schematic diagram of the BConv layer is shown in Figure 7.

Bconv modules of different colors represent convolution layers of different kernels (k represents the length and width of the kernel, s represents stride, o represents outchannel, i represents inchannel, where o = i represents outchannel = inchannel; o ≠ i represents outchannel has no correlation with inchannel). The first BConv module is a convolution module with k = 1 and s = 1, the second BConv module is a convolution module with k = 3 and s = 1, and the third BConv module is a convolution module with k = 3 and s = 2. The above Bconv modules with different colors mainly distinguish k and s, and do not distinguish the input and output channels.

Extended-ELAN based on ELAN is proposed in YOLOv7. The shortest and longest path of the gradient is controlled by an efficient long-range attention network so that the deep network can learn and converge more efficiently. The E-ELAN proposed by YOLOv7 uses methods such as expanding, shuffling, and merging cardinality to improve the learning ability of the network without destroying the original gradient path.

In terms of structure, E-ELAN only changes the architecture of the block itself but does not change the architecture of the transition layer. It uses group convolution to expand the channel and cardinality of the computing block and applies the same group to all computing blocks of the computing layer parameters and the number of channels. It then perform the following operations on the feature map output by each computing block: randomly scramble the g group parameters set by the feature map into g groups, and then connect them. Currently, the number of channels in each set of feature maps is the same as the number of channels in the original architecture. Finally, add the feature maps of the g group to merge the cardinality.

**Figure 8:**
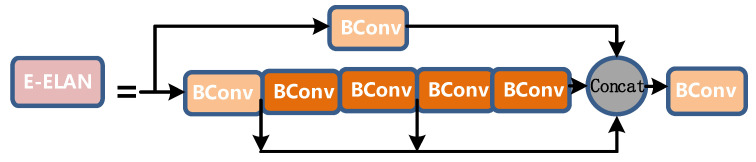
E-ELAN layer diagram.

The E-ELAN layer is also composed of different convolutions, as shown in Figure 8:

The length and width of the input and output of the entire E-ELAN layer are unchanged, and o = 2i on the channel, where 2i is spliced by the outputs of the four Bconv layers.

**Figure 9:**
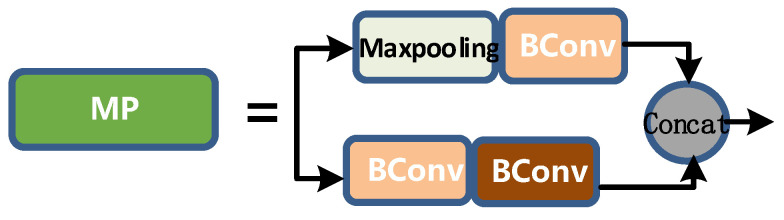
MP layer diagram.

MP layer is shown in Figure 9. Its input and output channels are the same. The output length and width are half of the input length and width. The upper branch first halves the length and width by max pooling, and then halves the channel by BConv. The lower branch halves the channel through the first BConv, then halves the length and width through the second Bconv, and then merges the upper and lower branches to get the corresponding output.

3. Head

**Figure 10:**
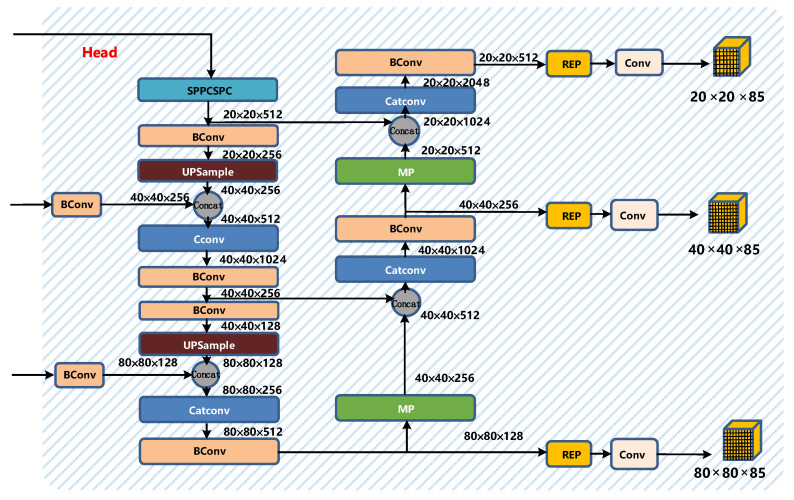
Head structure diagram of YOLOv7.

The head part of YOLOv7 is like the previous YOLOv4 and YOLOv5. The difference is that the CSP module in YOLOv5 is replaced by the E-ELAN module, and the downsampling module is changed to the MPConv layer. The entire head layer is composed of SPPCPC layers, several BConv layers, several MPConv layers, several Catconv layers, and RepVGG block layers that output three heads subsequently. A schematic diagram of the Head part of YOLOv7 is shown in Figure 10.

**Figure 11:**
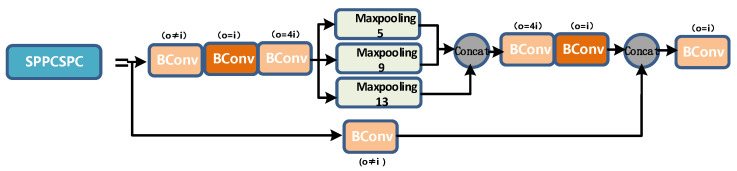
SPPCPC layer diagram.

The SPPCSPC layer is a module obtained by using the pyramid pooling operation and the CSP structure. It still contains many branches. Its total input will be divided into three segments in different branches. The output information is concat. A schematic diagram of the SPPCSPC layer is shown in Figure 11.

**Figure 12:**
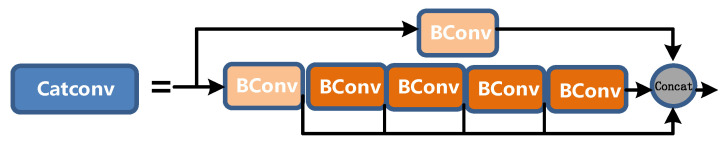
Catconv layer diagram.

The operation of the Catconv layer is the same as that of the E-ELAN layer, which also allows deeper networks to learn and converge more efficiently. A schematic diagram of the Catconv layer is shown in Figure 12.

**Figure 13:**
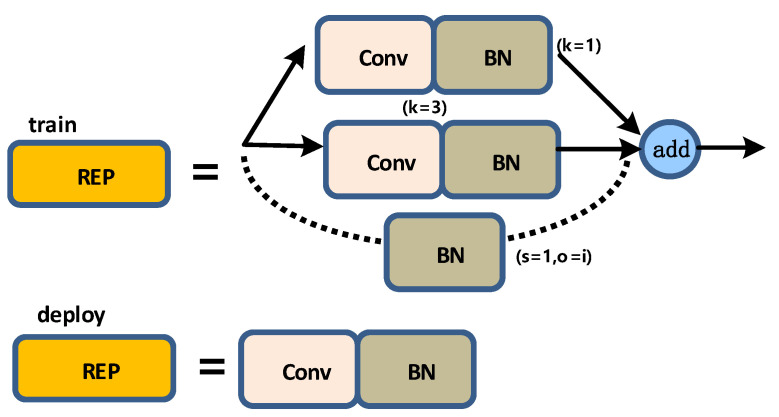
REP layer diagram.

The structure of the REP layer is not the same during training and deployment. During training, the REP layer adds a 1 × 1 convolution branch based on the 3 × 3 convolution. If the input and output channels and the dimensions of h and w are the same, a BN branch is added, and then the three branches are added for output; when deploying, to facilitate deployment, the parameters of the branch will be re-parameterized and then allocated to the main branch, and the 3 × 3 main branch convolution output will be taken. A schematic diagram of the structure of the REP layer is shown in Figure 13.

#### 2.2.2. Non-Parametric Attention Module

Sun Yat-Sen University proposed the conceptually simple and very effective attention module SimAM [57], which, unlike other attention modules, does not require additional parameters to compute 3D attention weights. This makes its predictions extremely efficient.

**Figure 14:**
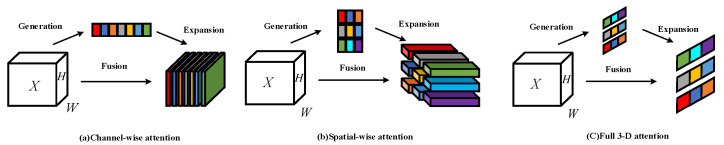
Comparison of the implementation process of different attention mechanisms.

Existing attention mechanism modules such as BAM and CBAM combine spatial attention and channel attention in parallel or series, respectively. However, attention is usually a collaborative way of working, not simply cobbled together in parallel or serially. Therefore, it is particularly important to unify the weights of the attention of the two mechanisms. Figure 14a represents the channel attention mechanism, which represents 1D attention, which treats different channels differently and treats all positions equally, and Figure 14b represents the spatial attention mechanism, which represents 2D attention, which pays attention to different positions. All channels are treated equally. Figure 14c represents the 3D attention mechanism, which can unify the weights of the channel and spatial attention.

In the implementation of the attention mechanism, the role of each neuron needs to be considered. In neuroscience, neurons that are rich in information tend to be particularly active. Moreover, such neurons usually inhibit surrounding neurons. To find such active neurons, the concept of the energy function is proposed, and the expression of the energy function is shown in Equation (8).

(8) $$e_{\delta }\left(w_{\delta },b_{\delta },y,o_{i}\right)={\left(y_{\delta }-\hat{\delta }\right)}^{2}+1/\left(M-1\right)\sum_{i=1}^{M-1}{\left(y_{o}-{\hat{o}}_{i}\right)}^{2}$$

where δ and $o_{i}$ refer to active neurons and other neurons between the single channel whose input feature is $X={\mathbb{R}}^{C\times H\times W}$, $\hat{\delta }=w_{\delta }\delta +b_{\delta }$ and ${\hat{o}}_{i}=w_{\delta }o_{i}+b_{\delta }$ are the linear transformation relationship between δ and $o_{i}$, i is the index in the spatial dimension, and M is the number of neurons on this channel. This calculation method is M=H×W, and $w_{\delta }$ and $b_{\delta }$ refer to the weight coefficient and bias coefficient of the transformation.

When this equation takes its minimum value, $\hat{\delta }$ is equal to $y_{\delta }$, and all other ${\hat{o}}_{i}$ are equal to $y_{o}$, where $y_{\delta }$ and $y_{o}$ are two different values.

Minimize the equation to obtain the linear relationship between the corresponding active neuron and other neurons. To simplify the calculation, binary labels are used for $y_{\delta }$ and $y_{o}$, and a regularization method is added to the equation. The expression of the final energy function is shown in Equation (9).

(9) $$e_{\delta }\left(w_{\delta },b_{\delta },y,o_{i}\right)=1/\left(M-1\right)\sum_{i=1}^{M-1}{\left(-1-\left(w_{\delta }o_{i}+b_{\delta }\right)\right)}^{2}+{\left(1-\left(w_{\delta }\delta +b_{\delta }\right)\right)}^{2}+\lambda w_{\delta }{}^{2}$$

In theory, each channel has the M=H×W energy function. The solution of the above equation is shown in Equation (10).

(10) $$w_{\delta }=-\frac{2\left(\delta -u_{\delta }\right)}{{\left(\delta -u_{\delta }\right)}^{2}+2\sigma_{\delta }^{2}+2\lambda }b_{\delta }=-\frac{1}{2}\left(\delta +u_{\delta }\right)w_{\delta }$$

Among these, $u_{\delta }=\frac{1}{M-1}\sum {}_{i=1}^{M-1}o_{i}$, and $\sigma_{\delta }^{2}=\frac{1}{M-1}{\sum {}_{i=1}^{M-1}\left(o_{i}-u_{\delta }\right)}^{2}$; therefore, the minimum energy solution can be represented by Equation (11).

(11) $$e_{\delta }^{*}=\frac{4\left({\hat{\sigma }}^{2}+\lambda \right)}{{\left(\delta -\hat{u}\right)}^{2}+2{\hat{\sigma }}^{2}+2\lambda }$$

The above equation means that the lower the energy, the more differentiated the neuron from the surrounding neurons, and the higher the degree of importance.

To verify whether the performance of the SimAM attention mechanism helps to improve the performance of the model, SE, CBAM, GC, ECA, and SRM attention mechanisms were selected for the control group to conduct a comparative experiment with SimAM, and the performance of YOLOv7 with different attention mechanisms was added. For example, see Table 2.

**Figure 15:**
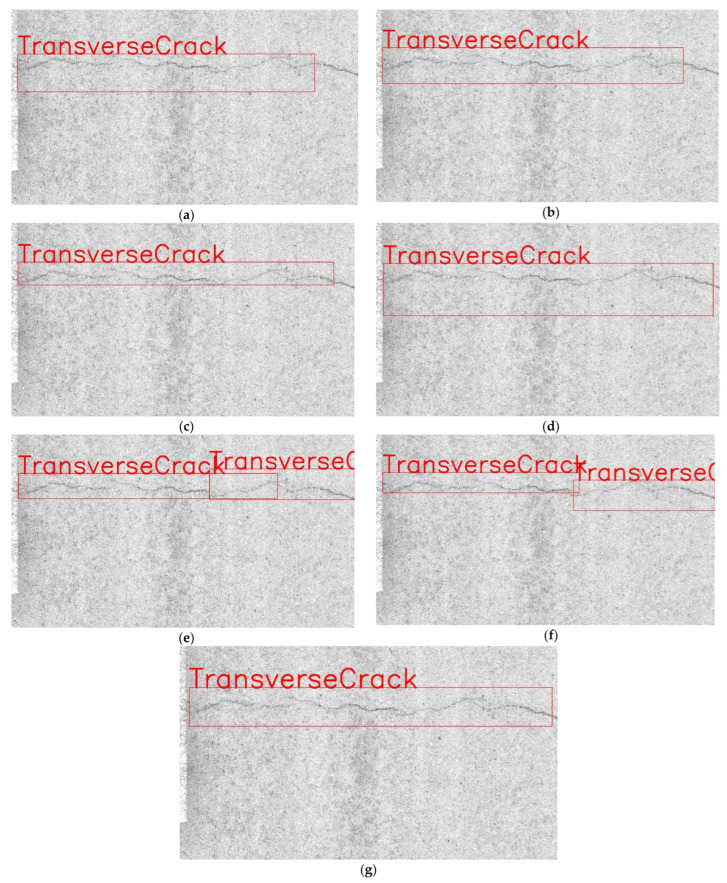
Detection results under different attention mechanisms. (**a**) Baseline. (**b**) Baseline + SE. (**c**) Baseline + CBAM. (**d**) Baseline + GC. (**e**) Baseline + ECA. (**f**) Baseline + SRM. (**g**) Baseline + SimAM.

To show the performance of YOLOv7 combined with SimAM attention mechanism more intuitively, this article combines the actual test results to make an intuitive display, and the actual test results are shown in Figure 15.

#### 2.2.3. Transformer

In 2017, the Transformer model with attention operation as the core was first proposed to provide a new deep network architecture for processing sequence features [58]. At present, Transformer has been successfully used in computer vision and other fields. The core of the Transformer model is the multi-head self-attention mechanism. The attention mechanism assigns high weight to high-value information, which is essentially an efficient allocation of information processing resources. Its adaptive attention weight distribution reflects the correlation between output data and input sequence features. A trainable neural network based on Transformer can complete the recognition task by building an encoder and a decoder.

In this paper, the Transformer encoder is constructed as the core of the classifier. The Transformer encoder is composed of N identical sub-modules (Transformer Block) stacked. As shown in the figure, the sub-module contains a multi-head attention layer (Multi-Head Attention) and a feedforward neural network. Layer (Feed-Forward Network) has two main parts: the introduction of residual connection (Residual Connection) and layer normalization (Layer Normalization, LN) to prevent gradient degradation and accelerate algorithm convergence.

**Figure 16:**
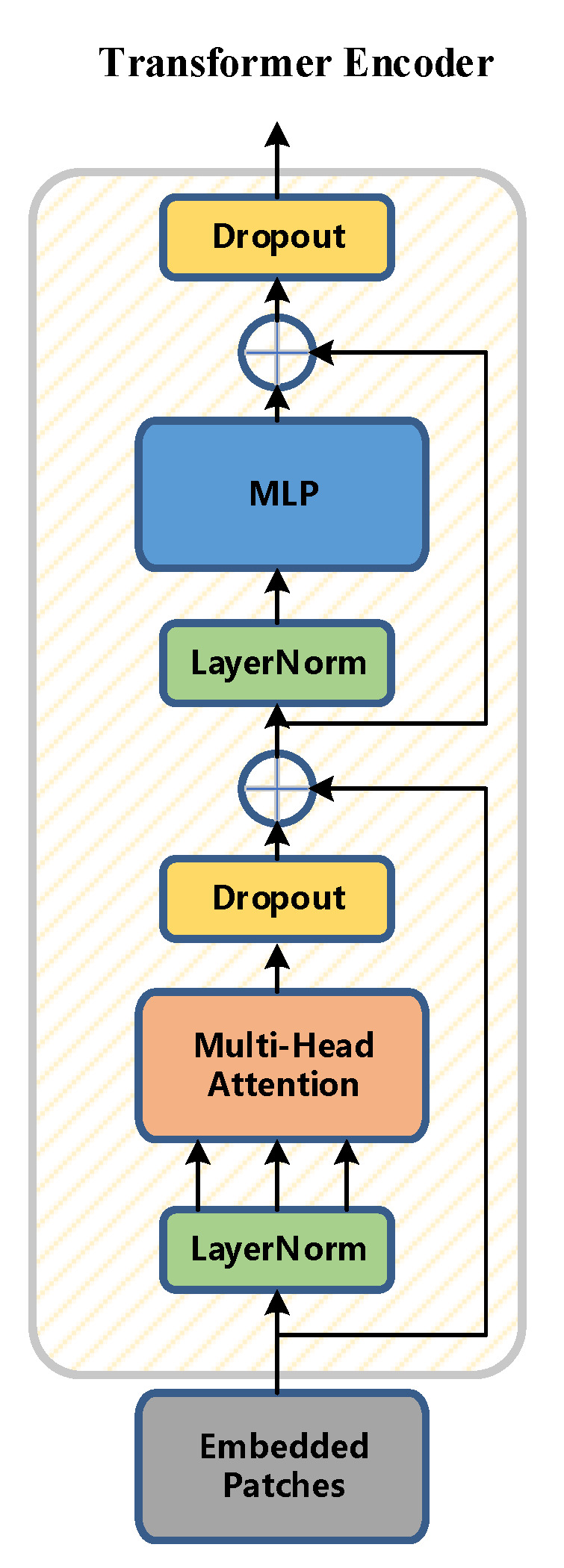
Transformer encoder diagram.

Compared with the traditional bottleneck block module, the Transformer encoder has a more powerful ability to collect information. Each Transformer encoder contains two parts: the first part is the multi-head attention layer, the second part is the MLP, and the Transformer encoder improves the ability to capture different raw information. A schematic diagram of the Transformer encoder is shown in Figure 16.

To verify whether the performance of adding the transformer encoder to the YOLOv7 network structure is improved, a set of ablation experiments were conducted, respectively, for the unimproved YOLOv7 network, the YOLOv7 network with the addition of the attention mechanism SimAM, and the addition of both the attention mechanism SimAM and the Transformer encoding. The YOLOv7 network of the device is used to verify Precision, Recall, and mAP@0.5. The results of the ablation experiments are shown in Table 3.

**Figure 17:**
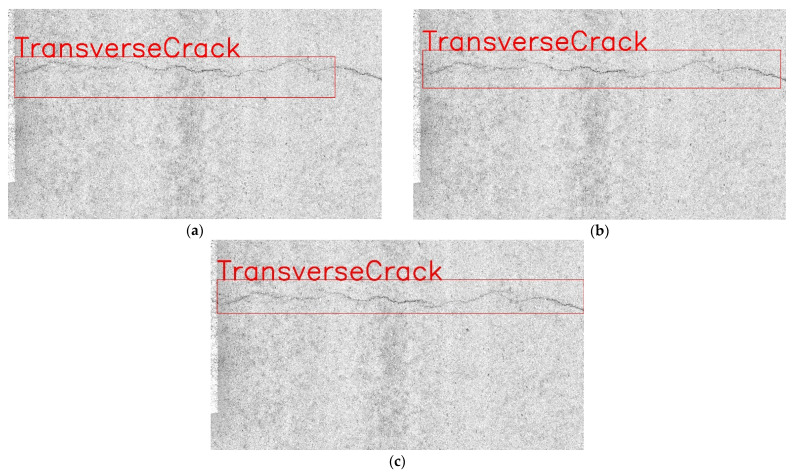
Detection results under different networks. (**a**) Baseline. (**b**) Baseline + SimAM. (**c**) Baseline + SimAM + Transformer.

To show the performance of YOLOv7 combined with Transformer more intuitively, this paper combines the actual test results for intuitive presentation. The actual test results are shown in Figure 17.

#### 2.2.4. Loss Function

The loss function needs to use traditional indicators such as distance, shape, and IoU in the process of calculating the mismatch between the real frame and the model predicted frame in the image. In addition, the direction of matching between the real frame and the predicted frame also needs to be matched, within consideration. None of the loss functions proposed and used so far consider the problem of orientation matching between the desired ground-truth box and the predicted box. Loss functions include IoU, GIoU [59], DIoU [60], CIoU [61], etc. In SIoU [62], the addition of this metric can greatly help the training convergence process and effect. To minimize distance-related variables, SIoU will predict as close as possible along the X or Y direction.

To verify whether the SIoU loss function is improved compared with other loss functions after combining with YOLOv7, multiple sets of control experiments were conducted. The results of the control experiments are shown in Table 4.

**Figure 18:**
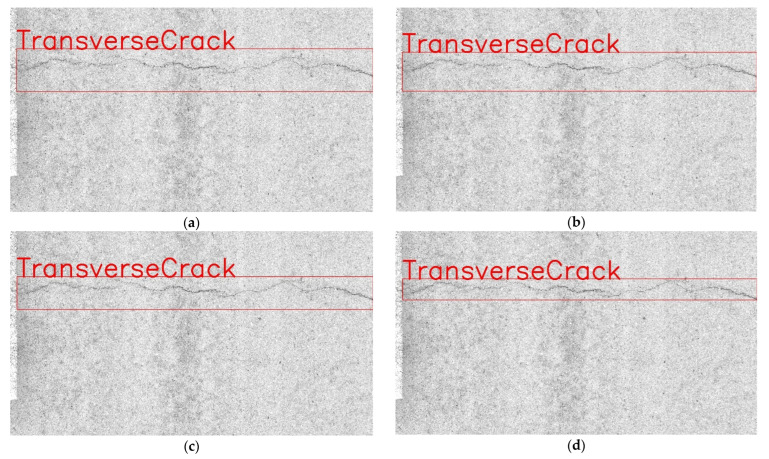
Detection results under different loss functions. (**a**) Baseline + SimAM + Transformer + GIoU. (**b**) Baseline + SimAM + Transformer + DIoU. (**c**) Baseline + SimAM + Transformer + CIoU. (**d**) Baseline + SimAM + Transformer + SIoU.

To show the performance of YOLOv7 in combination with different loss functions more intuitively, this paper combines the actual test results for intuitive display. The actual test results are shown in Figure 18.

#### 2.2.5. YOLO-SAMT Network Structure

**Figure 19:**
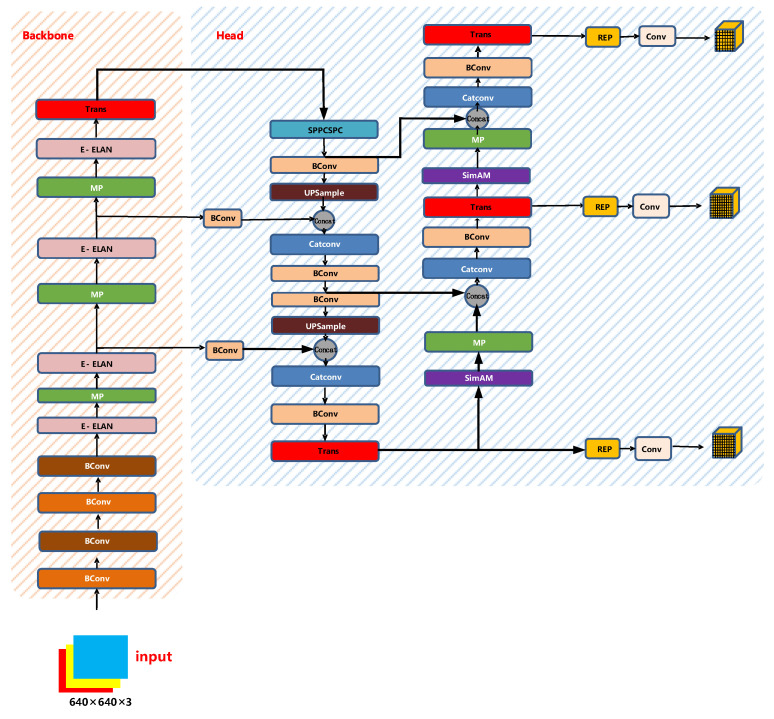
YOLO-SAMT network structure diagram.

After combining the parameter-less attention module SimAM, Transformer encoder, and SIoU loss function mentioned in the previous section, this paper proposes a new target detection model, which we name YOLO-SAMT, where the red module represents the added Transformer encoder, the purple module represents the added parameter-less attention module SimAM, and finally combines the SIoU loss function on the prediction side of the model. The network model of YOLO-SAMT is shown in Figure 19.

### 2.3. Crack Image Segmentation Based on Improved K-Means Clustering Algorithm

K-means [63] is used to classify the best class attribution of points by calculating the similarity of the distance between points. The k-means algorithm minimizes the objective function, clustering the data by separating the sample data into n classes of equal variance.

After preprocessing by guided filtering and the Retinex method, the crack target in the image can be highlighted, and the extraction of the crack feature is regarded as the binary classification of the image.

The two-dimensional crack image is expanded into one-dimensional samples, and F(x) is set as the gray value corresponding to the one-dimensional sample data point x of the image, $u_{j}^{i}$ represents the clustering center of class j after the i-th clustering, and $C_{j}^{i}$ represents the region where the samples divided into class j after the i-th clustering are located. The process of k-means clustering algorithm is as follows.

1. Initialize cluster center $u_{1}^{0},u_{2}^{0},\cdots ,u_{k}^{0}$;
2. Assume that a cluster center u is the nearest to F(x), and classify F(x) as the cluster center. The formula of this step is shown in Equation (12).
  (12) $$\left|F\left(x\right)-u\right|<\left|F\left(x\right)-u_{j}^{i}\right|,u\in \left\{u_{j}^{i},j=1,2,3,\cdots ,k\right\}$$
3. As shown in Equation (13), update the clustering center $u_{j}^{i}$, $F\left(x_{j}^{i}\right)$ is the gray value of sample $x_{j}^{i}$ in the $C_{j}^{i}$ region, and $n_{j}^{i}$ is the number of samples in the $C_{j}^{i}$ region;
  (13) $$m_{j}^{i}=\frac{1}{n_{j}^{i}}\sum_{0}^{n_{j}^{i}}F\left(x_{j}^{i}\right)$$
4. Calculate criterion function P of Equation (14), if P converges or end the iteration after reaching the maximum number of iterations C. Otherwise, go to step 2.
  (14) $$P=\sum_{j=1}^{k}\sum_{x_{j}^{i}\in C_{j}^{i}}{\left|F\left(x_{j}^{i}\right)-u_{j}^{i}\right|}^{2}$$

The initial cluster center point of k-means is randomly generated, and it is easy to generate errors if it is affected by noise or other outliers. Therefore, the following two methods are adopted for optimization.

1. Gaussian filtering is used to denoise the image to eliminate the interference of image outliers, and the filtered image is used as the initial image for extracting cracks;
2. When the image is classified into two, set k=2, take the gray value corresponding to the point with the most pixels in the grayscale histogram of the filtered image as $u_{1}^{0}$, and then calculate the point with the largest distance between the remaining sample data F(x) and $u_{1}^{0}$ as $u_{2}^{0}$. The expression of $u_{2}^{0}$ is shown in Equation (15).
  (15) $$u_{2}^{0}=\max\left\{F\left(x\right)-u_{1}^{0}\right\}$$

## 3. Experiment Results and Discussions

### 3.1. Data

The data in this paper come from Fuzhou, Xiamen, Longyan, and Quanzhou in Fujian Province. The main equipment used for data acquisition in this paper is the road multi-function inspection vehicle and vehicle-mounted laser scanner introduced in the United States.

**Figure 20:**
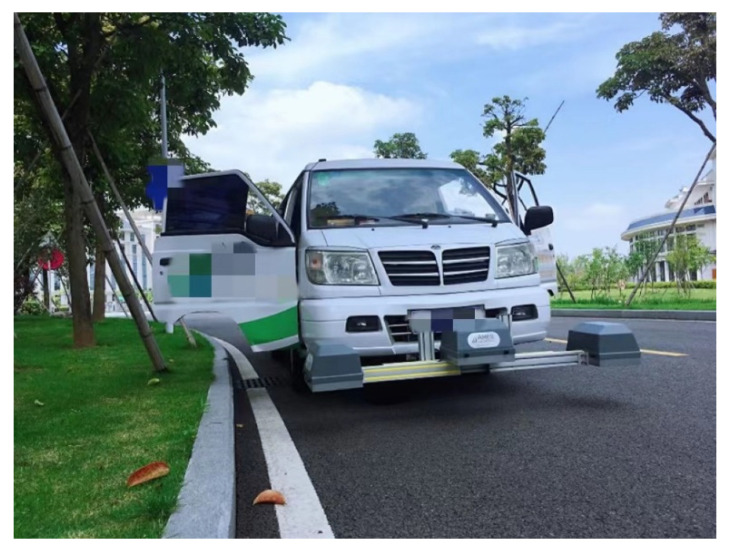
Road multi-function detection vehicle.

As the main data acquisition equipment, the road multi-function detection vehicle (DHDV) is composed of linear laser transmitters, line scan cameras, photoelectric encoders, and IMUs. The intelligent detection and identification of crack diseases provide data sources. The schematic diagram of the road multi-function detection vehicle is shown in Figure 20.

**Figure 21:**
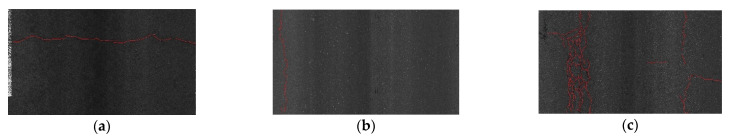
Diagram of crack disease sample: (**a**) Transverse crack; (**b**) Longitudinal crack; (**c**) Map crack.

The main research object of this paper is various asphalt pavement cracks. The schematic diagram of transverse cracks is shown in Figure 21a, the schematic diagram of longitudinal cracks is shown in Figure 21b, and the schematic diagram of map cracks is shown in Figure 21c.

The disease images of highway asphalt pavement in various counties and cities in Fujian Province were collected by DHDV, and the corresponding training set and validation set were constructed. The distribution of different types of crack samples in the data set is shown in Table 5.

### 3.2. Experimental Environment and Parameter Settings

Before the experiment, the pavement crack dataset was divided into two parts: a training set and a validation set according to the ratio of 7:3. The model parameters are updated in real-time during the training process. The complexity (model parameters and computation) of YOLO-SAMT proposed in this paper is comparable to that of Resnet50 and RepVGG-A2, and the experiments are more comparative. The hardware and software environments required for the experiment are shown in Table 6.

### 3.3. Evaluation Indicators

Precision (Pression, P), Recall (R), and mean average precision (mAP) provide an important reference index to evaluate the performance of the model.

By applying the evaluation index to test whether the crack disease location is accurate, two types of image results can be obtained, including pictures with cracks and pictures without cracks. When there are cracks in the image and the prediction result also shows cracks, we call it TP; when there are cracks in the image and the prediction result has no cracks, we call it FN; when there are no cracks in the image, and the prediction result shows cracks, we call it FP; when there is no crack in the image, and the prediction result also shows no crack, we call it TN. The above metrics are shown in Table 7.

In this study, the evaluation indicators precision rate (Precision, Pr), recall rate (Recall, Re), and F1-score were introduced to evaluate the performance of the crack identification and localization model and the crack segmentation model. The test image is input into the crack detection model; the number of TP, FP, and FN of the test results of the identification positioning model and the segmentation model is counted; and the evaluation indicators Pr, Re, and F1-score are finally calculated. Pr represents the proportion of all predicted positive samples that were correctly detected. Re represents the proportion of all actual positive samples that were successfully detected. The F1-score is an evaluation index to measure the comprehensive performance of the model. It objectively reflects the accuracy and recall rate of the model. The larger the F1-score, the stronger the model performance. The specific calculation formulas of the evaluation indicators are shown in Equations (16)–(18).

(16) $$\mathrm{Pr}=\frac{\mathrm{TP}}{\mathrm{TP}+\mathrm{FP}}$$

(17) Re=TPTP+FN

(18) $$F1-\mathrm{score}={\left(\frac{{\mathrm{Precision}}^{-1}+{\mathrm{Recall}}^{-1}}{2}\right)}^{-1}$$

### 3.4. Quantitative Analysis and Evaluation of Fracture Segmentation Model

To verify whether the fracture extraction results of the proposed algorithm meet the requirements, first of all, the real fractures extracted by the proposed algorithm are denoted as image A, and the fracture results extracted by the proposed algorithm are denoted as image B. Image C is obtained by performing or operating on image A and image B, and the number of pixels with 0 pixel value in image A, image B, and image C are respectively denoted as m(A), m(B), and m(C). The fracture feature coincidence degree is used to describe whether the fracture feature extraction algorithm is effective. The definition of value is shown in Equation (19).

(19) $$\mathrm{CR}=\frac{m\left(C\right)}{m\left(A\right)+m\left(B\right)-m\left(C\right)}$$

To ensure the integrity of crack extraction, if the CR value is greater than or equal to 80%, it is regarded as correct detection; otherwise, it is regarded as false detection.

To evaluate the accuracy of the algorithm in this paper, three parameters such as accuracy P, true rate T, and false positive rate F are used for analysis. The analysis equations are shown in Equations (20)–(22).

(20) $$P=\frac{\mathrm{TP}+\mathrm{TN}}{\mathrm{TP}+\mathrm{FN}+\mathrm{TN}+\mathrm{FP}}$$

(21) $$T=\frac{\mathrm{TP}}{\mathrm{TP}+\mathrm{FN}}$$

(22) $$F=\frac{\mathrm{FP}}{\mathrm{TN}+\mathrm{FP}}$$

where TP is the number of images that contain cracks, and the detected cracks meet the requirements (CR ≥ 80%); TN is the number of images whose images do not contain cracks and no cracks are detected at the same time; FP is number of the images that do not contain cracks but detect cracks; and FN is the number of images that contain cracks but are not detected, or the detection results do not meet the requirements (CR ≤ 80%).

### 3.5. Analysis

#### 3.5.1. Comparative Experiments of YOLO-SAMT with Other Models

To further verify the high efficiency of the YOLO-SAMT network architecture proposed in this paper, it is compared with other networks. These networks include the classic single-stage object detection network, the two-stage object detection network, and the advanced networks appearing in the references [30,31,34,35]. The training data and test data we selected are consistent with the previous section. To discuss the standard deviation of the results, in this section, we select three different roads to evaluate the performance of the models obtained by each network training. These three roads are Xiarong Expressway AK112, Shangjiao Expressway BK36, and Yonghang Expressway BK218. The number of standard cracks in these three roads is 342, 427, and 134, respectively. The index evaluated in this experiment is the accuracy of crack identification. After the test was completed, the average accuracy of each model for crack identification of three roads was counted. The test results are shown in Table 8.

Through the comparative experiments of different networks, taking the most representative network as an example, the YOLO-SAMT target detection model proposed in this paper is 13.96 percentage points higher than Faster RCNN in F1-score and 8.09 percentage points higher than YOLOv5s. In comparison with other excellent crack detection networks, the model proposed in this paper is 11.99 percentage points higher than the model proposed in [30]. From the above comparison experiments, the crack location method proposed in this paper has greater advantages than other network models. From the actual test results of the three test roads, the average recognition accuracy of the proposed method is 15.7 percentage points higher than that of Faster RCNN, and 7.6 percentage points higher than that of YOLOv5s. Compared with the method proposed in [30], the proposed model is 6.1 percentage points higher. It can be seen from the above comparative experiments that the crack location method proposed in this paper has greater advantages than other network models.

#### 3.5.2. Comparative Experiment of Fracture Segmentation Model

**Figure 22:**
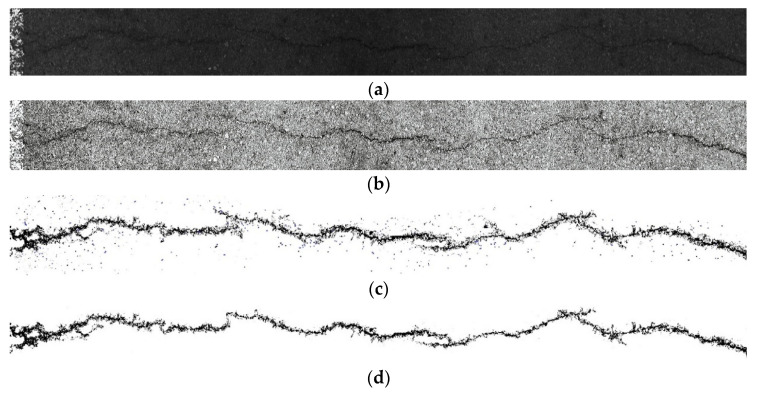
Comparative experiment of transverse crack segmentation. (**a**) original images, (**b**) image enhanced by guided filtering and Retinex method, (**c**) images generated by traditional k-means clustering algorithm, (**d**) ours.

**Figure 23:**
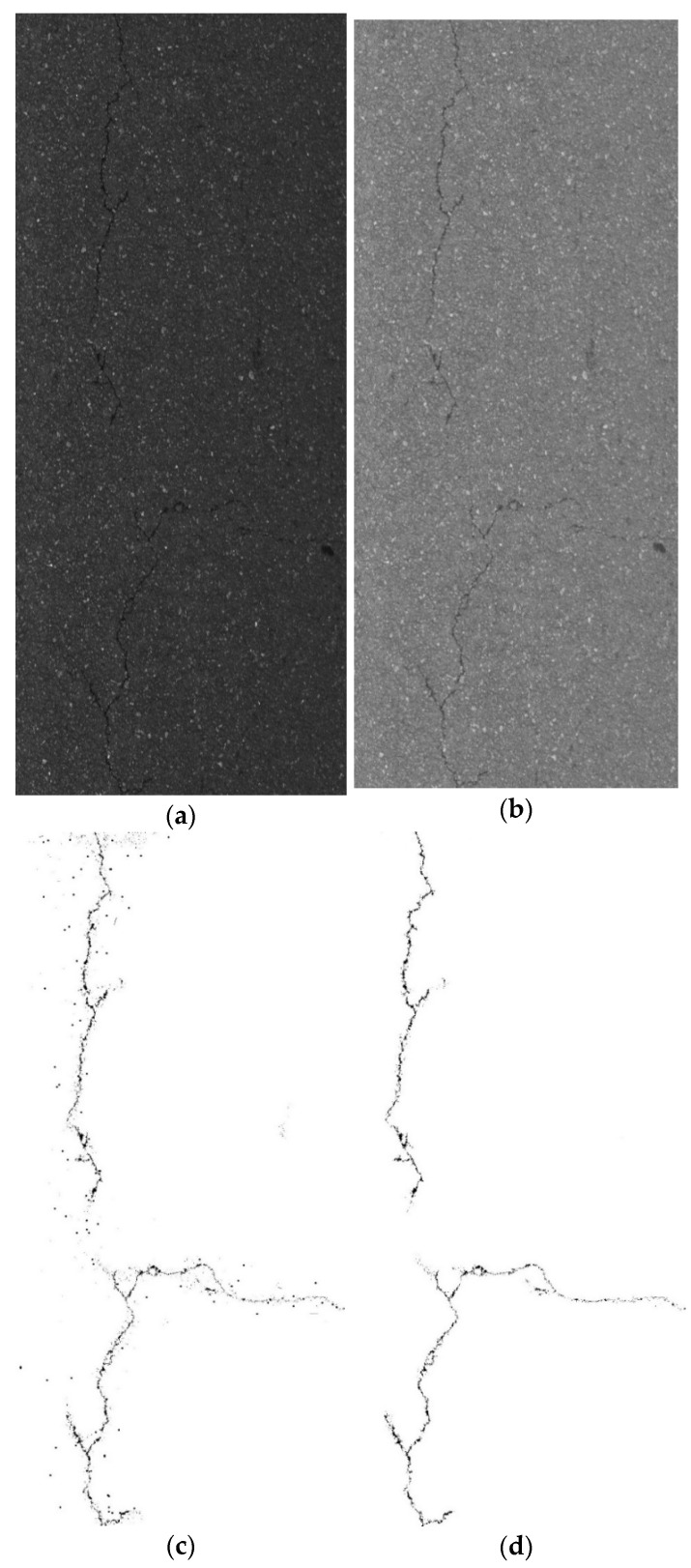
Longitudinal crack segmentation contrast experiment. (**a**) original images, (**b**) image enhanced by guided filtering and Retinex method, (**c**) images generated by traditional k-means clustering algorithm, (**d**) ours.

**Figure 24:**
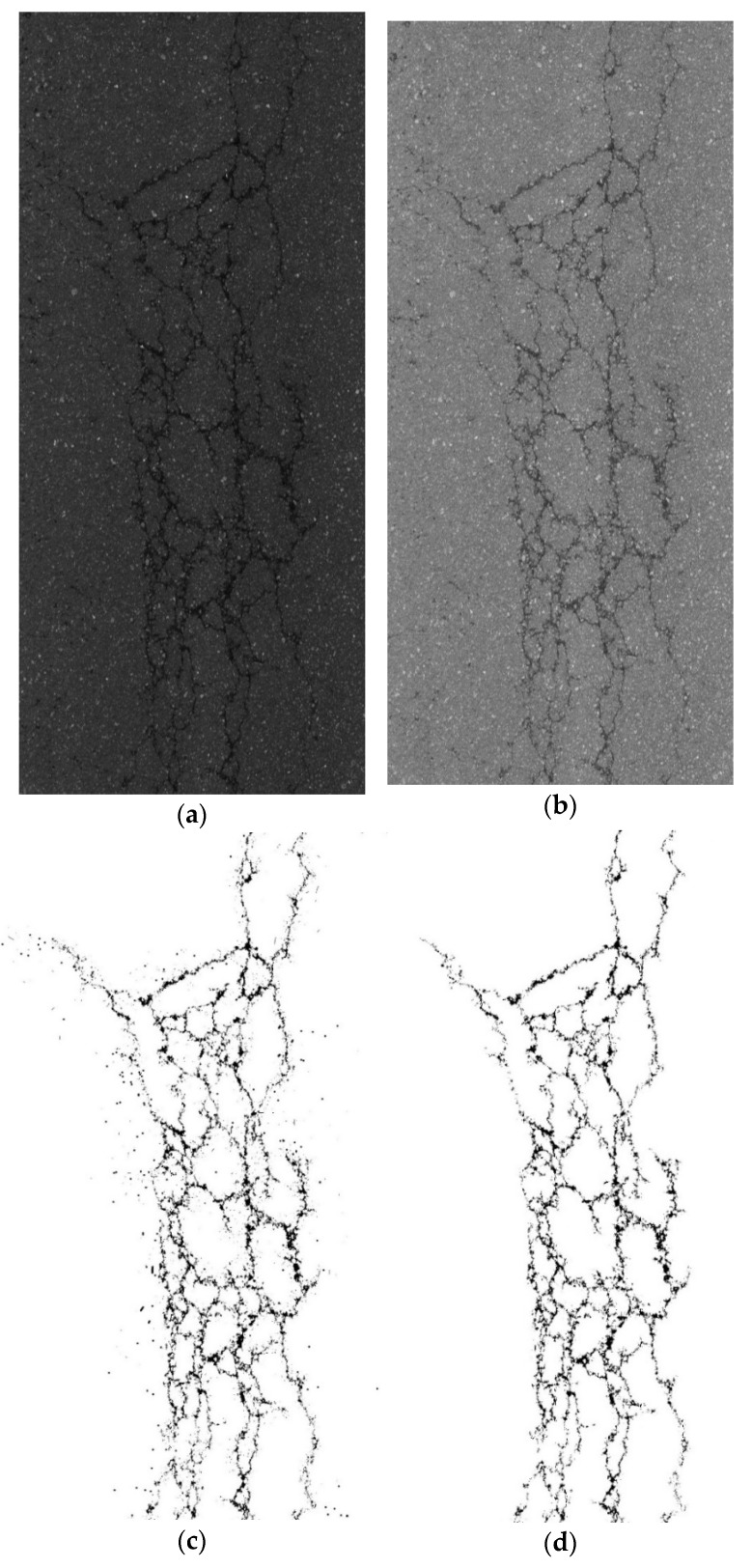
Comparative experiment of map crack segmentation. (**a**) original images, (**b**) image enhanced by guided filtering and Retinex method, (**c**) images generated by traditional k-means clustering algorithm, (**d**) ours.

After the YOLO-SAMT detection and positioning, the positioning area needs to be cropped. After cropping, the improved k-means clustering algorithm is used to extract the crack contour. To verify the superiority of the algorithm proposed in this paper, it is compared with the original image, the guided filtering and Retinex-enhanced image, and the traditional k-means clustering. The images generated by the algorithm are compared. In this section, transverse cracks, longitudinal cracks, and map cracks are selected for comparative experiments. The comparative experiments are shown in Figure 22, Figure 23 and Figure 24.

In this paper, 300 crack images are experimentally verified, including 100 transverse cracks, 100 longitudinal cracks, and 100 map cracks. Firstly, the crack features are manually extracted from the image as the real crack extraction result, then different preprocessing methods are used for preprocessing, then the method in this paper is used to extract cracks, and the detection ability of different preprocessing methods is calculated as shown in Table 9.

It can be seen from Table 9 that the detection accuracy of the crack extraction algorithm proposed in this paper is 96.67%, the true rate is 92.76%, and the false positive rate is only 1.25%. It is superior to other traditional image extraction algorithms.

## 4. Conclusions

This study combines traditional digital image processing technology and deep learning methods to achieve accurate positioning and segmentation of asphalt pavement cracks. Traditional digital image processing technologies such as MSR and MSRCR focus on adjusting the color or brightness of the image but ignore the denoising of the image. To solve this problem, this paper first performs a two-dimensional discrete wavelet transform on the image to obtain low-frequency coefficients and high-frequency coefficients of the wavelet. Then the wavelet low-frequency coefficients are processed by the combination of guided filtering and MSRCR algorithm, and the multi-scale information of the image is extracted on the premise of retaining the image color. Then the soft threshold filtering is used to denoise the wavelet high frequency coefficient, and the information entropy of the picture is improved as much as possible. After the data enhancement of the image, this paper also optimizes the target detection network, and further improves the positioning accuracy of the model by adding the attention mechanism and improving the loss function. When the crack disease location is completed, the corresponding image is clipped, and the improved k-means clustering algorithm is used to extract the contour of the crack, which greatly reduces the amount of calculation and improves the efficiency. The main contributions of this paper to the crack detection method are as follows: (1) A new image preprocessing method based on guided filtering and Retinex is proposed in this paper. Compared with traditional digital image detection technology, this method can eliminate the influence of external factors such as uneven illumination distribution, environmental noise and occlusion shadow on image quality. (2) In order to reduce the amount of calculation, targeted to extract the crack characteristics, this paper proposes an improved target detection algorithm based on the non-parametric attention module SimAM and Transformer. The purpose of this algorithm is to accurately locate the crack area in the image. Thus, the redundant calculation is reduced for the following crack contour feature extraction. Compared with the existing convolutional neural network and deep learning methods, this algorithm enhances the accuracy of frame selection for crack target area. (3) In this paper, the traditional k-means clustering algorithm has been improved through the Gaussian filter to eliminate image noise and optimize the image pixel value, which can provide a more accurate extraction of crack contour.

At the same time, the size of the parameters of the target detection model is also an important factor affecting the computational efficiency of the model. Although the accuracy of the model has been improved, the model compression still needs to be studied on a deeper level. Future research will focus on model parameter optimization.

## Figures and Tables

**Table 1:** Information entropy of crack defect images processed by various algorithms.

Method	Information Entropy
Original images	5.2525
OpenCV	5.1995
Gimp	5.4451
MSRCR	5.5761
SSR	5.6237
MSR	5.6700
Matlab	6.9788
MSRCP	7.5275
AutoMSRCR	7.6704
Ours	7.9835

**Table 2:** Performance comparison of YOLOv7 with different attention mechanisms.

Network/Index	Precision	Recall	mAP@0.5	Parameters	GFLOPS	Speed-GPU	Weight
Baseline	81.43	84.37	83.74	7,114,785	16.5	1.7	14.2
Baseline + SE	81.77	84.39	83.88	7,456,849	16.7	1.7	14.3
Baseline + CBAM	81.74	84.37	83.87	7,385,126	16.7	1.7	14.5
Baseline + GC	81.94	85.11	84.13	7,456,878	16.6	1.7	14.3
Baseline + ECA	82.10	85.77	84.79	7,998,452	16.9	1.5	14.9
Baseline + SRM	81.93	85.12	84.12	7,336,542	16.5	1.7	14.5
Baseline + SimAM	85.79	87.96	86.69	7,124,568	16.5	1.7	14.2

**Table 3:** Ablation experimental results.

Network/Index	Precision	Recall	mAP@0.5	Parameters	GFLOPS	Speed-GPU
Baseline	81.43	84.37	83.74	7,114,785	16.5	1.7
Baseline + SimAM	85.79	87.96	86.69	7,124,568	16.5	1.7
Baseline + SimAM + Transformer	87.99	88.11	88.06	7,139,546	16.5	1.7

**Table 4:** Performance comparison of different loss functions.

Network/Loss	mAP@0.5
Baseline + SimAM + Transformer + GIoU	88.12
Baseline + SimAM + Transformer + DIoU	88.34
Baseline + SimAM + Transformer + CIoU	88.03
Baseline + SimAM + Transformer + SIoU	89.16

**Table 5:** Distribution of training samples for each type of disease.

Data	Transverse Crack	Longitudinal Crack	Map Crack
Training set	1380	1391	1431
Validation set	614	622	583
Total	1994	2013	2014

**Table 6:** Hardware and software environment required for model training.

Surroundings	Category	Version
Hardware Environment	Operating system	Windows 10
	Graphics card configuration	GeForce RTX 3090Ti
Software Environment	Processor configuration	i7-8700 CPU
	Language	3.7
	Frame	Pytorch
	CUDA	11.1

**Table 7:** Target detection model evaluation indicators.

Forecast Result	Image Has Cracks	There Are No Cracks in the Image
Predicted as a crack	TP	FP
Predict no cracks	FN	TN

**Table 8:** Performance comparison of different networks.

Methods	F1-score(%)	(Correct/Total)			Average Accurate Rate
		AK112	BK134	BK150	
Standard	/	342/342	427/427	134/134	1
Two-stage	/	/	/	/	/
Faster R-CNN	75.47	251/342	301/427	75/134	0.666
Cascade R-CNN	75.99	253/342	311/427	71/134	0.666
Libra R-CNN	76.34	248/342	309/427	66/134	0.647
Grid R-CNN	76.21	261/342	300/427	61/134	0.640
Mask R-CNN	76.17	259/342	300/427	71/134	0.663
Dynamic R-CNN	76.13	250/342	303/427	77/134	0.672
One-stage	/	/	/	/	/
FCOS	76.81	257/342	301/427	69/134	0.675
FreeAnchor	76.43	255/342	311/427	66/134	0.656
RepPoints	76.56	249/342	309/427	73/134	0.666
PAA	76.77	259/342	307/427	75/137	0.679
ATSS	75.14	266/342	299/427	69/134	0.664
FoveaBox	73.49	240/342	309/427	77/134	0.667
FSAF	77.14	259/342	310/427	77/134	0.686
VFNet	75.99	255/342	311/427	71/134	0.668
SSD512	75.39	251/342	299/427	81/134	0.679
RetinaNet	76.41	266/342	313/427	81/134	0.705
YOLOv3	77.87	267/342	313/427	79/134	0.701
YOLOv4	78.31	270/342	316/427	88/134	0.729
YOLOv5s	81.34	281/342	323/427	89/134	0.747
Current research	/	/	/	/	/
Method [30]	77.44	283/342	333/427	91/134	0.762
Method [31]	82.63	288/342	318/427	79/134	0.726
Method [34]	84.50	281/342	316/427	88/134	0.740
Method [35]	86.50	289/342	334/427	90/134	0.766
Ours	89.43	299/342	359/427	101/134	0.823

**Table 9:** Comparison of detection ability of different methods.

Method	TP	TN	FP	FN	P/%	T/%	F/%
K-Means	231	37	9	23	89.33	86.19	19.57
Ours	269	21	3	7	96.67	92.76	1.25

## Data Availability

Not applicable.

## References

[1] Cheng H.-D., Chen J.-R., Glazier C., Chase S.B. (1996). Novel fuzzy logic approach to pavement distress detection. Nondestructive Evaluation of Bridges and Highways.

[2] Zuo Y., Wang G., Zuo C. Wavelet Packet Denoising for Pavement Surface Cracks Detection. Proceedings of the 2008 International Conference on Computational Intelligence and Security.

[3] Bhutani K.R., Battou A. (1995). An Application of Fuzzy Relations to Image Enhancement. Pattern Recognit. Lett..

[4] Kirschke K.R., Velinsky S.A. (1992). Histogram-based Approach for Automated Pavement-crack Sensing. J. Transp. Eng..

[5] Oliveira H., Correia L. Automatic Road Crack Segmentation Using Entropy and Image Dynamic Thresholding. Proceedings of the European Signal Processing Conference.

[6] Cheng H.D., Wang J., Hu Y.G., Glazier C., Shi X.J., Chen X.W. (2001). Novel Approach to Pavement Cracking Detection Based on Neural Network. Transp. Res. Rec..

[7] Cheng H.D., Shi X.J., Glazier C. (2003). Real-Time Image Thresholding Based on Sample Space Reduction and Interpolation Approach. J. Comput. Civ. Eng..

[8] Ge P., Chen Y., Wang G., Weng G. (2022). An Active Contour Model Driven by Adaptive Local Pre-Fitting Energy Function Based on Jeffreys Divergence for Image Segmentation. Expert Syst. Appl..

[9] Weng G., Dong B., Lei Y. (2021). A Level Set Method Based on Additive Bias Correction for Image Segmentation. Expert Syst. Appl..

[10] Ge P., Chen Y., Wang G., Weng G. (2022). A Hybrid Active Contour Model Based on Pre-Fitting Energy and Adaptive Functions for Fast Image Segmentation. Pattern Recognit. Lett..

[11] Tanaka S., Takanarita K., Okada H., Okazawa S., Kobayashi Y., Sudo M. (2013). A Study for Numerical Techniques for Fatigue Crack Propagation Analysis of Surface Crack in Welded Joints. J. Jpn. Soc. Nav. Archit. Ocean Eng..

[12] Talab A.M.A., Huang Z., Xi F., HaiMing L. (2016). Detection Crack in Image Using Otsu Method and Multiple Filtering in Image Processing Techniques. Optik.

[13] Salman M., Mathavan S., Kamal K., Rahman M. Pavement Crack Detection Using the Gabor Filter. Proceedings of the 16th International IEEE Conference on Intelligent Transportation Systems (ITSC 2013).

[14] Song H., Wang W., Wang F., Wu L., Wang Z. (2015). Pavement Crack Detection by Ridge Detection on Fractional Calculus and Dual-Thresholds. Int. J. Multimed. Ubiquitous Eng..

[15] Xu W., Radzieński M., Ostachowicz W., Cao M. (2013). Damage Detection in Plates Using Two-Dimensional Directional Gaussian Wavelets and Laser Scanned Operating Deflection Shapes. Struct. Health Monit..

[16] Montanari L., Basu B., Spagnoli A., Broderick B.M. (2015). A Padding Method to Reduce Edge Effects for Enhanced Damage Identification Using Wavelet Analysis. Mech. Syst. Signal Process..

[17] Andreaus U., Baragatti P., Casini P., Iacoviello D. (2017). Experimental Damage Evaluation of Open and Fatigue Cracks of Multi-Cracked Beams by Using Wavelet Transform of Static Response via Image Analysis: Experimental Damage Evaluation of Cracked Beams via Wavelets. Struct. Contr. Health Monit..

[18] Zhu L.F., Ke L.L., Xiang Y., Zhu X.Q. (2020). Free Vibration and Damage Identification of Cracked Functionally Graded Plates. Compos. Struct..

[19] Mallat S. (1999). Wavelet Packet and Local Cosine Bases. A Wavelet Tour of Signal Processing.

[20] Kumar R., Singh S.K. (2021). Crack Detection near the Ends of a Beam Using Wavelet Transform and High Resolution Beam Deflection Measurement. Eur. J. Mech. A Solids.

[21] Nigam R., Singh S.K. (2020). Crack Detection in a Beam Using Wavelet Transform and Photographic Measurements. Structures.

[22] Kumar R., Nigam R., Singh S.K. (2022). Selection of Suitable Mother Wavelet along with Vanishing Moment for the Effective Detection of Crack in a Beam. Mech. Syst. Signal Process..

[23] Kumar R., Singh S.K. (2022). A Variance-Based Approach for the Detection and Localization of Cracks in a Beam. Structures.

[24] Nasiri S., Khosravani M.R. (2022). Applications of Data-Driven Approaches in Prediction of Fatigue and Fracture. Mater. Today Commun..

[25] Myllyaho L., Raatikainen M., Männistö T., Mikkonen T., Nurminen J.K. (2021). Systematic Literature Review of Validation Methods for AI Systems. J. Syst. Softw..

[26] Nasiri S., Khosravani M.R., Weinberg K. (2017). Fracture Mechanics and Mechanical Fault Detection by Artificial Intelligence Methods: A Review. Eng. Fail. Anal..

[27] Das H.C., Parhi D.R. Application of Neural Network for Fault Diagnosis of Cracked Cantilever Beam. Proceedings of the 2009 World Congress on Nature & Biologically Inspired Computing (NaBIC).

[28] Das H.C., Parhi D.R. (2008). Online Fuzzy Logic Crack Detection of a Cantilever Beam. Int. J. Knowl. Based Intell. Eng. Syst..

[29] Oliveira H., Correia L. Supervised Strategies for Cracks Detection in Images of Road Pavement Flexible Surfaces. Proceedings of the European Signal Processing Conference.

[30] Lee B.J., Lee H.D. (2004). Position-invariant Neural Network for Digital Pavement Crack Analysis. Comput. Aided Civ. Infrastruct. Eng..

[31] Zhang A., Wang K.C.P., Li B., Yang E., Dai X., Peng Y., Fei Y., Liu Y., Li J.Q., Chen C. (2017). Automated Pixel-Level Pavement Crack Detection on 3D Asphalt Surfaces Using a Deep-Learning Network: Pixel-Level Pavement Crack Detection on 3D Asphalt Surfaces. Comput. Aided Civ. Infrastruct. Eng..

[32] Han C., Ma T., Huyan J., Huang X., Zhang Y. (2021). CrackW-Net: A Novel Pavement Crack Image Segmentation Convolutional Neural Network. IEEE Trans. Intell. Transp. Syst..

[33] Li D., Xie Q., Gong X., Yu Z., Xu J., Sun Y., Wang J. (2021). Automatic Defect Detection of Metro Tunnel Surfaces Using a Vision-Based Inspection System. Adv. Eng. Inform..

[34] Huyan J., Li W., Tighe S., Zhai J.Z., Xu Z.C., Chen Y. (2019). Detection of Sealed and Unsealed Cracks with Complex Back-Grounds Using I (A) Deep Convolutional Neural Network. Autom. Constr..

[35] Malini A., Priyadharshini P., Sabeena S. (2021). An Automatic Assessment of Road Condition from Aerial Imagery Using Modified VGG Architecture in Faster R-CNN Framework. J. Intell. Fuzzy Syst..

[36] Cha Y.-J., Choi W., Büyüköztürk O. (2017). Deep Learning-Based Crack Damage Detection Using Convolutional Neural Networks: Deep Learning-Based Crack Damage Detection Using CNNs. Comput. Aided Civ. Infrastruct. Eng..

[37] Mogalapalli H., Abburi M., Nithya B., Bandreddi S.K. (2021). Classical-Quantum Transfer Learning for Image Classifica-Tion. SN Comput. Sci..

[38] Pang J., Zhang H., Feng C., Li L. (2020). Research on Crack Segmentation Method of Hydro-Junction Project Based on Target Detection Network. KSCE J. Civ. Eng..

[39] Sekar A., Perumal V. (2021). Automatic Road Crack Detection and Classification Using Multitasking Faster RCNN. J. Intell. Fuzzy Syst..

[40] Jobson D.J., Rahman Z., Woodell G.A. (1997). Properties and Performance of a Center/Surround Retinex. IEEE Trans. Image Process..

[41] Jobson D.J., Rahman Z.U., Woodell G.A. (1997). A Multiscale Retinex for Bridging the Gap between Color Images and the Hu-Man Observation of Scenes. IEEE Trans. Image Process.

[42] Fu X., Zeng D., Huang Y., Zhang X.P., Ding X. A Weighted Variational Model for Simultaneous Reflectance and Illu-Mination Estimation. Proceedings of the IEEE Conference on Computer Vision and Pattern Recognition.

[43] Guo X., Li Y., Ling H. (2016). LIME: Low-Light Image Enhancement via Illumination Map Estimation. IEEE Trans. Image Process..

[44] Gedraite E.S., Hadad M. Investigation on the Effect of a Gaussian Blur in Image Filtering and Segmentation. Proceedings of the Elmar International Symposium Electron.

[45] Magnier B., Montesinos P., Diep D. (2011). Ridges and Valleys Detection in Images Using Difference of Rotating Half Smooth-Ing Filters. International Conference on Advanced Concepts for Intelligent Vision Systems.

[46] Liu J., Xu C., Zhao Y. Improvement of Facial Expression Recognition Based on Filtering and Certainty Check. Proceedings of the 2021 International Conference on Electronic Information Engineering and Computer Science (EIECS).

[47] Wu G., Luo S., Yang Z. (2020). Optimal Weighted Bilateral Filter with Dual-range Kernel for Gaussian Noise Removal. IET Image Process..

[48] Shi Z., Zhu M.M., Guo B., Zhao M., Zhang C. (2018). Nighttime Low Illumination Image Enhancement with Single Image Using Bright/Dark Channel Prior. EURASIP J. Image Video Process..

[49] Gonzalez R.C., Wintz P. (1979). Digital Image Processing.

[50] He K., Sun J., Tang X. (2010). Guided Image Filtering. Computer Vision—ECCV 2010.

[51] Lin Y., Xu X., Ye C. (2021). Adaptive Stochastic Resonance Quantified by a Novel Evaluation Index for Rotating Machinery Fault Diagnosis. Measurement.

[52] Redmon J., Divvala S., Girshick R., Farhadi A. (2015). You Only Look Once: Unified, Real-Time Object Detection. arXiv.

[53] Redmon J., Farhadi A. (2016). YOLO9000: Better, Faster, Stronger. arXiv.

[54] Redmon J., Farhadi A. (2018). YOLOv3: An Incremental Improvement. arXiv.

[55] Bochkovskiy A., Wang C.-Y., Liao H.-Y.M. (2020). YOLOv4: Optimal Speed and Accuracy of Object Detection. arXiv.

[56] Wang C.-Y., Bochkovskiy A., Liao H.-Y.M. (2022). YOLOv7: Trainable Bag-of-Freebies Sets New State-of-the-Art for Real-Time Object Detectors. arXiv.

[57] Yang L., Zhang R.Y., Li L., Xie X. Simam: A Simple, Parameter-Free Attention Module for Convolutional Neural Net-Works. Proceedings of the 38th International Conference on Machine Learning.

[58] Chen H., Jiang X., Dai Y. (2022). Shift Pose: A Lightweight Transformer-like Neural Network for Human Pose Estimation. Sensors.

[59] Zhang H., An L., Chu V.W., Stow D.A., Liu X., Ding Q. (2021). Learning Adjustable Reduced Downsampling Network for Small Object Detection in Urban Environments. Remote Sens..

[60] Lv Y., Ai Z., Chen M., Gong X., Wang Y., Lu Z. (2022). High-Resolution Drone Detection Based on Background Difference and SAG-YOLOv5s. Sensors.

[61] Shang D., Zhang J., Zhou K., Wang T., Qi J. (2022). Research on the Application of Visual Recognition in the Engine Room of Intelligent Ships. Sensors.

[62] Han G., Li T., Li Q., Zhao F., Zhang M., Wang R., Yuan Q., Liu K., Qin L. (2022). Improved Algorithm for Insulator and Its Defect Detection Based on YOLOX. Sensors.

[63] Li X., Xu S., Jiang T., Wang Y., Ma Y., Liu Y. (2022). POI Recommendation Method of Neural Matrix Factorization Integrating Auxiliary Attribute Information. Mathematics.

